# One-Step Synthesis of Diamine-Functionalized Graphene Quantum Dots from Graphene Oxide and Their Chelating and Antioxidant Activities

**DOI:** 10.3390/nano10010104

**Published:** 2020-01-04

**Authors:** Rabeb El-Hnayn, Laetitia Canabady-Rochelle, Christophe Desmarets, Lavinia Balan, Hervé Rinnert, Olivier Joubert, Ghouti Medjahdi, Hafedh Ben Ouada, Raphaël Schneider

**Affiliations:** 1 Laboratoire des Interfaces et des Matériaux Avancés, Faculté des Sciences de Monastir, Avenue de l’Environnement, 5019 Monastir, Tunisia; lahnayen1986@gmail.com (R.E.-H.); hafedhbenouada@gmail.com (H.B.O.); 2 Laboratoire Réactions et Génie des Procédés, LRGP, Université de Lorraine, CNRS, F-54000 Nancy, France; Laetitia.canabady-rochelle@univ-lorraine.fr; 3 Institut Parisien de Chimie Moléculaire UMR-CNRS 8232, Sorbonne Université, 4 Place Jussieu, 75252 Paris CEDEX 5, France; christophe.desmarets@sorbonne-universite.fr; 4 Institut de Science des Matériaux de Mulhouse (IS2M), CNRS, UMR 7361, 15 rue Jean Starcky, 68093 Mulhouse, France; lavinia.balan@cnrs-orleans.fr; 5 CEMHTI-UPR3079 CNRS, Site Haute Température, 1D avenue de la Recherche Scientifique, 45071 Orléans, France; 6 Institut Jean Lamour, Université de Lorraine, CNRS, IJL, 54506 Vandoeuvre-lès-Nancy CEDEX, France; herve.rinnert@univ-lorraine.fr (H.R.); olivier.joubert@univ-lorraine.fr (O.J.); ghouti.medjahdi@univ-lorraine.fr (G.M.)

**Keywords:** graphene quantum dots, 2,2’-(ethylenedioxy)bis(ethylamine), optical properties, redox-active nanoparticles

## Abstract

2,2’-(Ethylenedioxy)bis(ethylamine)-functionalized graphene quantum dots (GQDs) were prepared under mild conditions from graphene oxide (GO) via oxidative fragmentation. The as-prepared GQDs have an average diameter of ca. 4 nm, possess good colloidal stability, and emit strong green-yellow light with a photoluminescence (PL) quantum yield of 22% upon excitation at 375 nm. We also demonstrated that the GQDs exhibit high photostability and the PL intensity is poorly affected while tuning the pH from 1 to 8. Finally, GQDs can be used to chelate Fe(II) and Cu(II) cations, scavenge radicals, and reduce Fe(III) into Fe(II). These chelating and reducing properties that associate to the low cytotoxicity of GQDs show that these nanoparticles are of high interest as antioxidants for health applications.

## 1. Introduction

Graphene quantum dots (GQDs) are planar nanocrystals in the 3–10 nm range that are composed of one or a few layers of graphene and the hexagonal lattice of sp^2^ carbons is edged with functional groups, like carboxylic acids or alcohols. GQDs exhibit a high surface-to-volume ratio and quantum confinement, surface defects, and edges effects being involved in the photoluminescence (PL) of GQDs [1,2,3,4,5]. Indeed, the PL emission of GQDs originates from isolated sp^2^ domains that are surrounded by tetravalent carbons and it depends on the size, the shape, the edge configuration, the surface defects, and on the functional groups present at the nanoparticles surface [6,7,8,9]. The bandgap of GQDs can be tuned by the control of the conjugation length and/or the surface chemistry [10,11,12,13,14,15,16]. GQDs have recently attracted high interest as alternatives to metal-based semiconductor QDs for numerous applications, like bio-imaging [17,18] and drug delivery [19,20], light-emitting diodes [21,22,23], optoelectronic devices [24,25], electrocatalysis [26,27], or biosensors [28,29,30], due to these unique optical properties, high chemical and photo-stability, low toxicity, low cost, and easy functionalization.

In recent years, the functionalization of GQDs with amine groups has gained interest in tuning the electronic and optical properties of GQDs due to the strong interactions between the nitrogen atoms and the graphene network (orbital interactions between the nonbounded pair of electrons of N with the HOMO and LUMO orbitals at GQDs allowing for a narrowing of the bandgap) [26]. The synthesis of amine-functionalized GQDs is generally conducted at high temperature while using ammonia [11,28,31,32,33,34] or urea [35], which react with epoxides, alcohols, and carboxylic acids groups that are present at the surface of oxidized graphene sheets to yield GQDs edge-terminated with primary amine functions.

Primary amine-assisted cutting of graphene oxide (GO) and the associated functionalization of GO sheets has only been scarcely investigated for the preparation of GQDs while using alkyl or aromatic amines. Blue-emitting GQDs modified with poly(ethylene imine) (PEI) or *N*-doped can be prepared by hydrothermal treatment at high temperature (150–230 °C) of GO or exfoliated graphite flakes with PEI [36,37]. Hydrophobic amines were also used to increase the solubility of GQDs in organic solvents because the hydrophilic properties of GQDs limit their applications in electronic and energy storage devices. This was either achieved by treating GO with thionyl chloride SOCl_2_ to transform carboxylic acid functions into acid chlorides, followed by the nucleophilic attack of the amine or via the amidative cutting of GO at 200 °C, followed by a reduction of the graphene network using hydrazine [38,39]. As previously indicated, amines, like phenazine, o-phenylenediamine, or benzoimidazole were used to prepare GQDs valuable for the development of optoelectronic devices, while ethylene diamine or 1,3-propanediamine were used to engineer GQDs-based bio-imaging probes, due to the strong interactions between the N atoms and GQDs HOMO and LUMO energy levels and the associated bandgap decrease [40] [25,41]. Finally, dimethylamino functionalized GQDs could be prepared by solvothermal treatment at 200 °C of GO in DMF used as solvent and nitrogen source [17].

Here, we report first a mild and one-step synthesis of 2,2’-(ethylenedioxy)bis(ethylamine)-functionalized GQDs via amine-assisted cutting of GO while using hydrogen peroxide H_2_O_2_ at 80 °C. The green-yellow emitting GQDs that were obtained exhibit a high PL quantum yield of 22% and a PL emission wavelength dependent on the excitation wavelength. Next, we demonstrate that GQDs could trap Fe(II) and Cu(II) ions and scavenge radicals like the 2,2’-azino-bis(3-ethylbenzothiazoline-6-sulfonic acid) radical (ABTS^•+^) due to their reducing activity, owing to the radical scavenging properties of the sp^2^-carbon domains [42,43,44,45,46,47,48] and the chelation ability of both oxygen-containing functions and of the 2,2’-(ethylenedioxy)bis(ethylamine) moieties [49,50,51].

## 2. Materials and Methods

### 2.1. Materials

Graphite powder (size < 150 µm, 99.99%, Sigma, Saint-Quentin Fallavier, France), potassium permanganate KMnO_4_ (>99%, Sigma), hydrogen peroxide H_2_O_2_ (30%, VWR Chemicals, Briare, France), sodium nitrate NaNO_3_ (>99%, Sigma), sulfuric acid H_2_SO_4_ (reagent grade), 2,2’-(ethylenedioxy)bis(ethylamine) (98%, Sigma), ferrous chloride FeCl_2_ (99.9%, Sigma), ferrozine (>99%, Sigma), potassium ferricyanide K_3_Fe(CN)_6_ (>99%, Sigma), ethylenediaminetetraacetic acid (EDTA) (>99%, Sigma), L-carnosine (99%, Sigma), murexide (ACS reagent, Sigma), copper sulphate CuSO_4_ (>99%, Sigma), Trolox*^®^* (6-hydroxy-2,5,7,8-tetramethylchroman-2-carboxylic acid, >98%, Sigma), potassium persulfate K_2_S_2_O_8_ (>99%, Sigma), trichloroacetic acid (>99%, ThermoFisher Scientific, Illkirch-Graffenstaden, France), and 2,2’-azino-bis(3-ethylbenzothiazoline-6-sulfonic acid) (ABTS) (99%, Sigma) were used as received without further purification. All of the solutions were prepared while using Milli-Q water (18.2 MΩ.cm, Millipore) as solvent.

### 2.2. Synthesis of Graphene Oxide (GO)

Graphite (2.0 g) was mixed with sodium nitrate NaNO_3_ (1 g, 11.76 mmol) and the mixture cooled to 5 °C while using an ice bath. Subsequently, concentrated sulfuric acid (50 mL) was added dropwise while maintaining the temperature below 20 °C. The mixture was further stirred for 30 min at a temperature below 20 °C. Potassium permanganate KMnO_4_ (300 mg, 1.90 mmol) was added portionwise over 30 min while keeping the temperature below 20 °C. A second portion of KMnO_4_ (7 g, 44.29 mmol) was added and the mixture was stirred 1 h at room temperature. The temperature was then raised to 35 °C and the mixture further stirred for 2 h. Next, the mixture was cooled to room temperature and H_2_O (90 mL) was slowly added (the temperature increased to 70 °C) and the mixture was stirred for 15 min. A mixture of H_2_O_2_ (7 mL) and H_2_O (55 mL) was then added to reduce the residual KMnO_4_ to soluble manganese ions. The obtained dispersion was filtered, washed by centrifugation with 5% HCl (4000 rpm, 10 min, 4 × 20 mL), and then with H_2_O (4000 rpm, 10 min, 5 × 20 mL), and finally dried at 60 °C for 24 h to obtain a brownish graphite oxide powder.

The graphite oxide powder was dispersed in water (1 mg/mL) and the suspension was ultra-sonicated for 1 h while using a sonication probe (frequency of 20 kHz, amplitude of 25%) to obtain a graphene oxide (GO) suspension. Finally, the suspension was centrifuged at 5000 rpm for 15 min to collect GO pellets, which were then dried at 60 °C for 24 h before use.

### 2.3. Synthesis of Graphene Quantum Dots (GQDs)

GO (20 mg) was dispersed in water (5 mL) under sonication. 2,2’-(ethylenedioxy)bis(ethylamine) (1.5 mL, 10.27 mmol) in H_2_O (8.5 mL) and H_2_O_2_ (40 mL, 30% solution in H_2_O) were then added and the pH of the solution adjusted to 7 while using 1 M HCl. The mixture was then transferred into a three-necked flask and heated at 80 °C under inert atmosphere for 8 h. After cooling to room temperature, the mixture was filtered using a filter membrane (0.4 µm) to remove the largest particles and then concentrated in vacuum to a final volume of ca. 15 mL. The dark yellow solution of GQDs obtained was dialyzed while using a 1 kDa MWCO membrane for 24 h to remove the unreacted starting materials, salts, and small fragments. A typical synthesis affords ca. 15 mg of GQDs. GQDs were stored at 4 °C either dispersed in water or as a powder after evaporation of water.

### 2.4. GQDs Characterization

Transmission electron microscopy (TEM) images were taken by placing a drop of the particles dispersed in water onto a carbon film-supported copper grid. The samples were studied using a CM200 instrument operating at 200 kV (Philips, Suresnes, France). The X-ray powder diffraction (XRD) diagrams were measured while using Panalytical X’Pert Pro MPD diffractometer (Malvern, Orsay, France) using Cu Kα radiation. The powder samples were placed on a silicon zero-background sample holder and the XRD patterns were recorded at room temperature using Cu K_α_ radiation (λ = 0.15418 nm). 

All of the optical measurements were performed at room temperature (20 ± 1 °C) under ambient conditions. The FT-IR spectra were recorded on a Brucker ALPHA spectrometer (Bruker, Palaiseau, France) that was equipped with a Platinum ATR accessory with a single diamond crystal. The absorption spectra were recorded on a Thermo Scientific Evolution 220 UV-visible spectrophotometer (Thermo Fisher, Illkirch-Graffenstaden, France). Photoluminescence emission spectra were measured on a Fluoromax-4 Jobin Yvon spectrofluorimeter (HORIBA Jobin Yvon, Longjumeau, France). The PL spectra were spectrally corrected and PL QYs were determined relative to Rhodamine 6G in ethanol (PL QY = 94%).

For the time resolved photoluminescence (TR-PL) experiments, the GQDs were pumped by the 355 nm line of a frequency-tripled YAG (yttrium aluminium garnet):Nd laser. The laser pulse frequency, energy, and duration were typically equal to 10 Hz, 50 µJ, and 10 ns, respectively. The PL signal was analyzed by a monochromator that was equipped with a 600 grooves/mm grating and by a photomultiplier tube cooled at 190 K. The rise time of the detector is equal to around 3 ns.

The cyclic voltammetry (CV) was carried out with an Autolab PGSTAT 100 workstation (Metrohm) that was controlled by an external PC and while using a standard three-electrode setup at 25 °C. The working vitreous carbon electrode (3 mm diameter) was polished with 6 μm diamond paste. A Pt wired served as counter electrode while a calomel electrode (SCE) was employed as reference electrode and then separated from the bulk by a double bridge. Electrochemical grade tetra-butylammoniumhexafluorophosphate employed as the supporting electrolyte in commercially anhydrous DMF at a concentration of 10^−1^ mol L^−1^. Typically, the solution of GQDs was purged with argon flow 10 min, and the voltammogram was recorded at ambient temperature with a scan rate of 20 mV/s.

### 2.5. Biocompatibility

The human THP-1 monocytic cell line was obtained from American Type Culture Collection (ATCC, TIB-202TM, Manassas, VA, USA). The cells were grown under standard conditions (37 °C, 5% CO_2_) in RPMI 1640 medium that was supplemented with 10% of heat-inactivated foetal calf serum, 100 U/mL of penicillin, 100 µg/mL of streptomycin, and 0.25 µg/mL of amphotericin. The cells were split every three days. 

The cell viability assay was analysed while using the WST-1 assay (Roche, 11644807001, Mannheim, Germany), according to the manufacturer’s protocol [52]. The THP-1 cells were seeded at 5 × 10^4^ cells/mL in 96-well plates and then exposed to different concentrations of GQDs. After 24 h, the WST-1 reagent was added in each well and the cells were incubated at 37 °C for 2 h. The absorbance of the solution was determined at 480 nm on a microplates reader (BioRad-iMARK) to determine the IC_50_. Each experiment is carried out on three independent replicates.

### 2.6. Antioxidant Properties of GQDs

All of the experiments were replicated five times and the various means and the standard errors of means were calculated.

#### 2.6.1. Iron(II)-Chelating Activity

The ability of GQDs to chelate Fe(II) was measured according to ref [53] and then adapted to microplate [54]. A volume of 7.5 µL of a 2 mM FeCl_2_ solution was added to 277.5 µL of GQDs used at various concentrations. After 3 min of incubation at room temperature, the reaction was inhibited by the addition of 15 µL of a 5 mM ferrozine solution. After 10 min, the absorbance was measured at 562 nm and then corrected by the absorbance of the microplate. EDTA (solutions from 0 to 100 µM) and carnosine (solutions from 0 to 1 mM) were used as references and positive controls. The experiment was replicated five times for GQDs or the references. A decrease in absorbance corresponds to an increase in Fe(II)-chelating capacity. The ability of a sample to chelate ferrous Fe(II) ions was defined, as follows:

(1) $$\mathrm{Iron}\;\mathrm{chelating}\;\mathrm{capacity}\left(\%\right)=\frac{A_{0}-A_{s}}{A_{0}}\times 100$$

where A_0_ and A_S_ are the blank and the sample absorbance, respectively.

#### 2.6.2. Copper(II)-Chelating Activity

The antioxidant capacity that was determined by Cu(II) chelation was measured by spectrophotometry while using murexide as a colorimetric indicator. This test was adapted from literature, as described in ref [55]. In the presence of a copper(II)-chelating sample, the copper-murexide complex dissociates, then forms free murexide in solution and a chelator-copper complex. The absorbance was measured at 485 nm and 520 nm to monitor the murexide-copper complex, and free murexide in solution, respectively. The absorbance ratio A_485_/A_520_ was considered to be proportional to the amount of free Cu(II) concentration in solution [56,57].

The copper(II)-chelating activity of GQDs was investigated between 0 and 2 mg/mL. Briefly, 143 μL of GQDs solutions were deposited in a well, to which 143 μL of a Cu(II) solution (3 mM CuSO_4_) prepared in hexamine buffer (10 mM hexamine, 10 mM KCl, pH 5) were added. Subsequently, 14 μL of a 1 mM aqueous solution of murexide was added to the mixture. After 3 min of incubation at room temperature, the absorbance was measured at 485 nm and 520 nm. The absorbance of the microplate at such wavelengths was subtracted to these former absorbance values.

For the blank, a value of A_0_ was measured corresponding to the absorbance of the reaction mixture without GQDs, with hexamine buffer replacing the latter. The copper chelation of GQDs was determined on the basis of the carnosine calibration (0–40 mM) as reference.

The copper chelation capacity was calculated according to Equation (2).

(2) $${\mathrm{Cu}}^{2+}\;\mathrm{Chelating}\;\mathrm{activity}\;(\%)\;=\;\frac{\left(\frac{A_{485}}{A_{520}}\right)0\;-\left(\frac{A_{485}}{A_{520}}\right)S}{\left(\frac{A_{485}}{A_{520}}\right)0}\times 100$$

where A_0_ is the blank absorbance and A_S_ the sample absorbance.

#### 2.6.3. Radical Scavenging Activity

The radical scavenging activity of GQDs was studied by the TEAC method that was described in refs [54,58,59]. Briefly, a ABTS^•+^ solution (7 mM) was prepared 24 h before the experiment by a reaction of ABTS with K_2_S_2_O_8_ (2.45 mM) in 4 mM-PBS (pH 7.4) and then stored in the dark. The ABTS^•+^ solution was diluted in PBS just prior the experiment to reach an absorbance of 0.70 ± 0.01 at 734 nm. Volumes of 150 µL of GQDs (from 0 to 2 mg/mL) or of the Trolox*^®^* solution (from 0 to 60 µM), both being prepared in 4 mM-PBS, were incubated with 150 µL of the ABTS^•+^ diluted solution at ambient temperature for 10 min. Subsequently, the spectrophotometry measurements were performed with a microplate reader at 734 nm (Multiskan™ GO instrument, ThermoFisher Scientific, Illkirch-Graffenstaden, France).

The radical scavenging activity was calculated, as follows:

(3) $$\mathrm{Radical}\;\mathrm{scavenging}\;\mathrm{activity}\;\left(\%\right)=\frac{A_{0}-A_{S}}{A_{0}}\times 100$$

where A_0_ is the blank absorbance (reaction medium with sample volume replaced by phosphate buffer 4 mM) and A_S_ is the absorbance of the remaining radical in the presence of GQDs or reference sample. Each absorbance (A_0_ or A_s_) was corrected by the absorbance of the void microplate that was measured at λ = 734 nm.

#### 2.6.4. Reducing Activity

The method described in ref [54,60] was adapted to microvolumes while using a 96-well microplate reader to determine the reducing capacity of GQDs. First, the GQDs solution at 2 mg/mL in ultrapure water was dialyzed (membrane cut-off 1 kDa) against 200 mM phosphate buffer (pH 6.6) during 24 h. Subsequently, the solutions of GQDs (0–2 mg/mL), as well as ascorbate used as reference (0–60 µM), were prepared and diluted in 200 mM phosphate buffer, pH 6.6. For a 70-µL diluted sample solution, 35 µL of a 1% (w/v) K_3_Fe(CN)_6_ solution were added and incubation was carried out for 20 min. at 50 °C. Afterwards, 135 µL of ultrapure water, 33 µL of a 10% (w/v) trichloroacetic acid solution, and 27 µL of a 0.1% (w/v) FeCl_3_ solution were added to the reaction medium and then incubated for 10 min. at room temperature. Note that all of these former solutions were prepared in 200 mM phosphate buffer, pH 6.6. Subsequently, absorbances were measured at 700 nm. The reducing capacity was expressed in percentage (%) and calculated, as follows:

(4) $$\mathrm{Reducing}\;\mathrm{capacity}\;\left(\%\right)=100\;-(\frac{{(A}_{0\;}-{\;A}_{S})}{A_{0}}\times 100)$$

where A_0_ is the absorbance of a 66 µM Prussian blue solution measured in the same reaction medium free of ascorbate and A_s_ is the sample absorbance. Each absorbance (A_0_ or A_s_) was corrected by the absorbance of the void microplate that was measured at λ = 700 nm.

## 3. Results

### 3.1. Synthesis and Structure of GQDs

**Figure 1:**
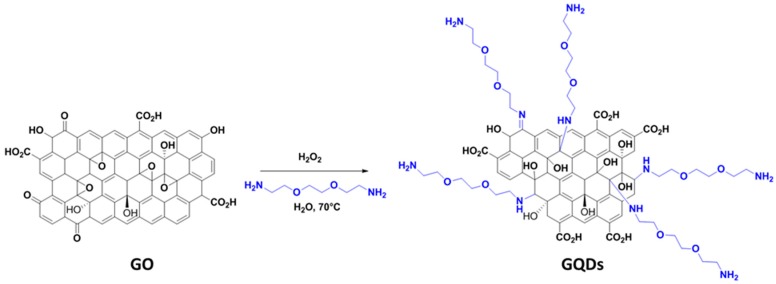
Schematic illustration of graphene quantum dots (GQDs) synthesis from graphene oxide (GO).

GQDs were produced by oxidative fragmentation of GO while using 2,2’-(ethylenedioxy)bis(ethylamine) and H_2_O_2_ (Figure 1). In the preliminary experiments, we found that heating of GO with H_2_O_2_ or of GO with 2,2’-(ethylenedioxy)bis(ethylamine) does not afford GQDs, indicating the synergistic action of H_2_O_2_ and of the diamine for the oxidative fragmentation of GO. GO is broken down into small particles by H_2_O_2_ and simultaneously the diamine reacts with the epoxide and carbonyl functions present at the surface of GO to yield 1,2-aminoalcohol and imine functions during the reaction. During the synthesis of GQDs, the reaction solution changed from colorless to dark yellow. In complementary experiments not described here, other amines and diamines, like butylamine, ethylene diamine, or 1,6-diaminohexane, were successfully used for the production of GQDs, which indicated that the synthetic process that was developed in this work is fairly general and not limited to 2,2’-(ethylenedioxy)bis(ethylamine). However, the latter affords GQDs exhibiting a higher PL QY that the other amines tested (*vide infra*).

**Figure 2:**
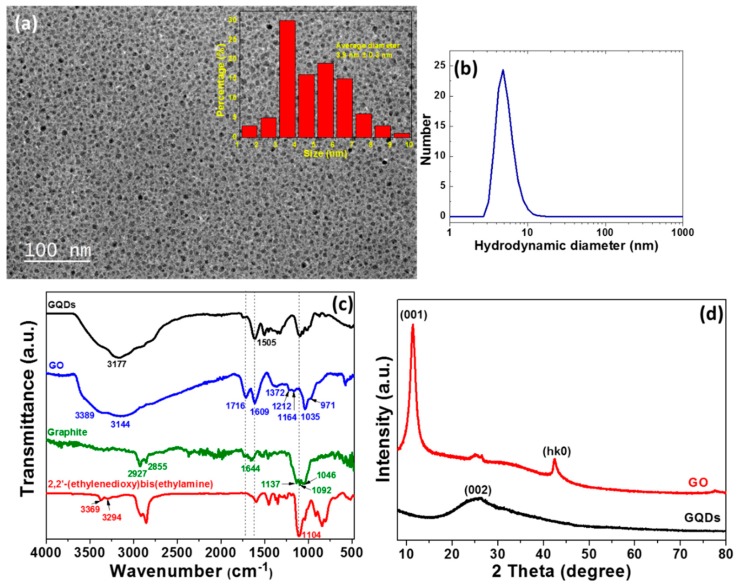
(**a**) Transmission electron microscopy (TEM) image and (**b**) dynamic light scattering (DLS) size data of GQDs. The inset of (**a**) is the TEM size distribution determined from the average of ca. 100 measurements. DLS measurements (mean ± s.d.) were from *N* = 3 independent experiments. (**c**) FT-IR spectra of 2,2’-(ethylenedioxy)bis(ethylamine), graphite, GO and of GQDs. (**d**) X-ray powder diffraction (XRD) patterns of GO and GQDs.

Figure 2a provides a typical transmission electron microscopy (TEM) image of produced GQDs. GQDs exhibit a spherical/ellipsoidal morphology with a uniform size distribution (inset of Figure 2a). Their average diameter is of 3.9 ± 0.3 nm. A high-resolution TEM image could not be recorded due to the high sensitivity of GQDs under the electron beam of the microscope, but their crystallinity was demonstrated by X-ray diffraction (XRD) (*vide infra*). Dynamic light scattering (DLS) analysis of GQDs that were dispersed in aqueous solution confirms that the dots have a narrow size distribution (4.8 ± 2.3 nm) (Figure 2b).

FT-IR spectroscopy, X-ray diffraction (XRD), and ^1^H and ^13^C NMR also characterized GQDs. The FT-IR spectrum of starting graphite shows the stretching ν vibrations of C-H bonds (2927 and 2855 cm^−1^), C=C bonds (1644 cm^−1^), and of C-O bonds (1137, 1092, and 1046 cm^−1^) originating from the light oxidation of graphite [61] (Figure 2c). Upon oxidation and exfoliation, strong signals that were located at 3389 cm^−1^ (ν OH), 1716 cm^−1^ (ν C=O carbonyl), 1609 cm^−1^ (ν C=O of CO_2_H and ν C=C), 1212 cm^−1^ (ν C-OH), and 1035 cm^−1^ (ν C-O-C epoxide) can be observed in the FT-IR of GO, which agrees with previous reports [62]. The carbonyl and epoxide signals disappear after the oxidative cutting of GO in the presence of 2,2’-(ethylenedioxy)bis(ethylamine). In the meantime, the C-O-C stretching of the diamine located at 1104 cm^-1^ appears in the FT-IR spectrum of GQDs.

The XRD pattern of powdered GO shows a strong and sharp peak that was located at 11.3° corresponding to the (001) reflection of GO with a d-spacing of 0.775 nm (Figure 2d). This increased d-spacing as compared to graphite (0.335 nm along the c-axis direction) originates from the functionalization of the sides and of the edges of graphene sheets by oxygenated groups (carboxylic acids, alcohols, epoxides) and from water molecules that were embedded between hydrophilic GO sheets. The weak signal at 2θ = 42.47° corresponds to a (hk0) plane of GO [63]. The reflections of GO disappear after a reaction with 2,2’-(ethylenedioxy)bis(ethylamine) and H_2_O_2_ (GQDs) and they are replaced by a broad reflection at 2θ = 25.37°, which is the typical graphene (002) signature [64].

The ^1^H NMR spectrum of GQDs that were dispersed in D_2_O show strong signals between 3.1 and 4.5 ppm for CH_2_ groups linked to O or N. A signal for ethylenic H is also observed at 8.11 ppm (Figure S1). In the ^13^C NMR spectrum, the presence of carboxylic acid and imine functions (181, 177, and 172 ppm), ethylenic carbons (signals between 130 and 103 ppm), C-O functions (signals between 76 and 57 ppm), and finally of C-N bonds could also be identified (Figure S2).

**Figure 3:**
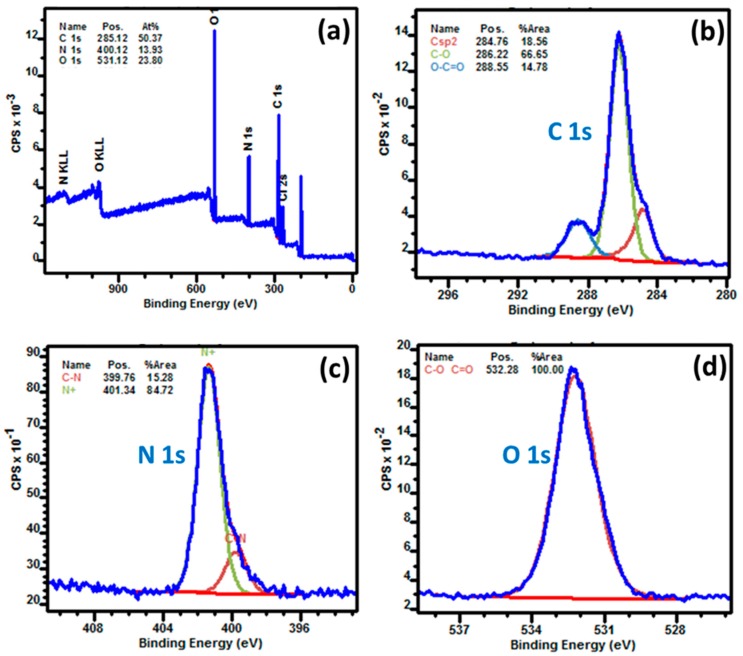
XPS data of GQDs (**a**) full-scan XPS spectrum, (**b**) C 1s spectrum, (**c**) N 1s spectrum, and (**d**) O 1s spectrum.

The chemical composition of GQDs and the chemical state of the elements were further ascertained by X-ray photoelectron spectroscopy (XPS). The survey spectrum shows that GQDs were only composed of C, O, and N (Figure 3a). The C/O and C/N atomic ratios are 2.1 and 3.6, respectively. Three types of C atoms could be detected in the C1s high resolution XPS spectrum: graphitic C-C and C=C (284.76 eV), C-O functions (288.22 eV), and C=O and CO_2_H groups (288.55 eV) (Figure 3b). However, the later two functions may be present in GQDs due to the weak energy difference between C-C or C=C and C-N or C=N functions [65]. As shown in Figure 3c, two N 1s signals located at 399.76 eV (C-N and C=N bonds) and at 401.34 eV (protonated NH_3_^+^ functions linked to carbon atoms), further confirming the covalent anchorage of 2,2’-(ethylenedioxy)bis(ethylamine) at the surface of GQDs either via C-N or C=N bonds. The high resolution O 1s spectrum shows a single component that is located at 532.38 eV that corresponds to the C-O and C=O functions (Figure 3d).

### 3.2. Optical Properties

**Figure 4:**
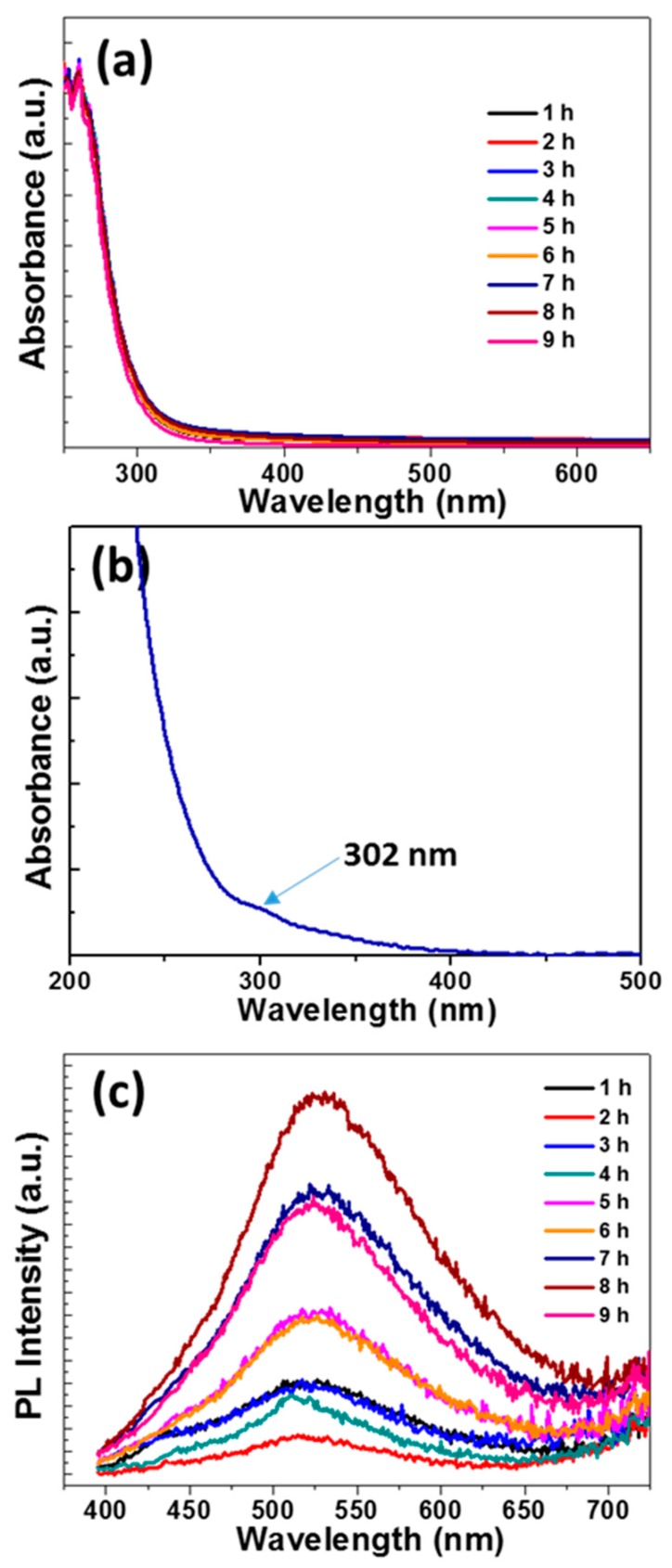
(**a**) Temporal evolution of UV-vis absorption spectra during the synthesis of GQDs and (**b**) magnification of the 200–500 nm range showing the n → π* transition at 302 nm. (**c**) Temporal evolution of photoluminescence (PL) emission spectra during the synthesis of GQDs (λ_ex_ = 375 nm).

Figure 4a,c provides the temporal evolution of UV-vis absorption and PL emission spectra during the synthesis of GQDs. All of the samples exhibit a strong absorption in the UV region (251 and 261 nm) corresponding to the π → π* transition of C=C bonds in the graphene structure [66]. A weaker absorption at 302 nm that corresponds to the n → π* of C=O and/or of C=N bonds could also be detected (Figure 4b). PL appears ca. after 1 h of reaction and it reaches its maximum value after 8 h of heating at 80 °C, after which the PL intensity decreases. A slight shift of the PL emission maximum is observed during the synthesis of GQDs from 517 after 1 h to 527 nm after 8 h, indicating that the molecular structure of GQDs changed in the course of the reaction. However, the PL peak shape and the full-width at half-maximum (fwhm) remained almost unchanged.

**Figure 5:**
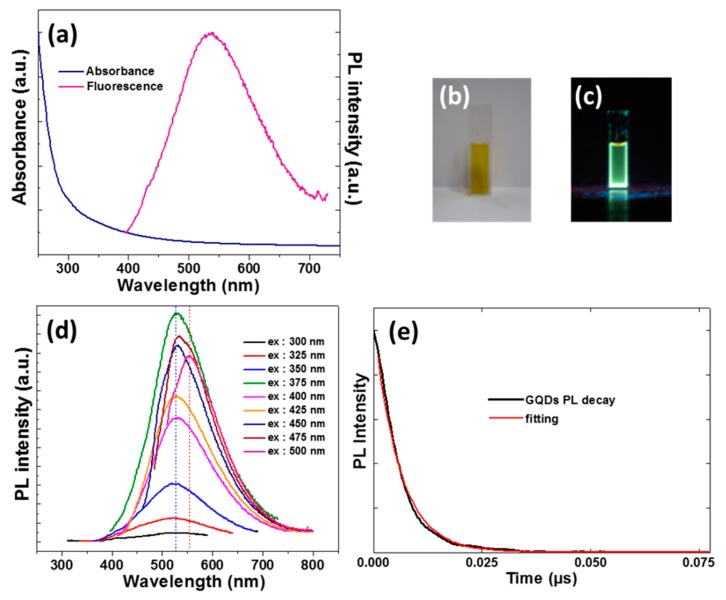
(**a**) UV-vis absorption and PL emission spectra of GQDs (ex = 375 nm). Photographs of an aqueous dispersion of GQDs (**b**) under room light and (**c**) under UV light. (**d**) PL emission spectra of GQDs as a function of the excitation wavelength ranging from 300 to 500 nm with an increment of 25 nm. (**e)** PL decay curve of GQDs at the emission maximum (530 nm) after excitation at 355 nm.

Figure 5a shows the UV-visible absorption and the PL emission (λ_ex_ = 375 nm) of GQDs that were obtained after purification by dialysis. GQDs are well dispersed in water and they emit a green-yellow fluorescence under UV light illumination (Figure 5b,c). The PL emission spectra of GQDs exhibit the characteristic feature of luminescent graphene-based nanomaterials, in which the PL emission maximum is shifted to lower energy when the excitation wavelength increases (Figure 5d and Figure S3). This shift originates from the optical selection of different surface defect states near the Fermi level of GQDs. However, the shift that is observed with GQDs is not gradually bathochromically shifted with the increase of the excitation wavelength, as commonly observed with graphene-based QDs [67]. The PL emission maximum is located at 528 nm for excitation wavelength between 300 and 450 nm. A second PL emission centered at 554 nm appears while using the 475 nm excitation wavelength and this contribution becomes the dominant emission peak when exciting GQDs at 500 nm. These results demonstrate that there are two electron transition pathways in the band structure of GQDs (green emission for λ_ex_ < 450 nm and yellow emission for λ_ex_ > 450 nm). The highest PL intensity is observed while using the 375 nm excitation wavelength and the PL QY in water is 22%.

The recombination dynamics of GQDs after excitation at 355 nm and at the PL maximum were also investigated. Figure 5e shows the PL decay profile and the corresponding fitting. Bi-exponential decays are usually observed for GQDs, the fast decay (τ < 1 ns) originates from the intrinsic states (exciton states and localized states), while the slow decay (1 < τ < 10 ns) corresponds to defect states [32,68]. The fast decay could not be measured with our equipment, but the slow decay could be fitted to a single-exponential function with a time constant of 5.205 ns, indicating that the recombination mainly occurs via specific states (for example, S_1_ → S_0_). The PL lifetime measured for GQDs is in the same range than that measured for amino-functionalized GQDs that were described in the literature [31,32]. The relatively long PL decay lifetime of GQDs and their high PL QY suggest that these nanoparticles are suitable for biological applications.

**Figure 6:**
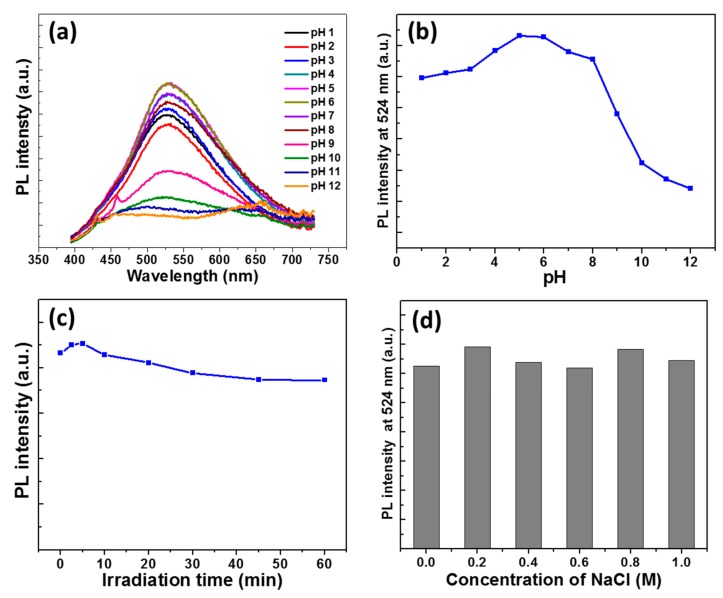
(**a**) PL emission spectra of GQDs when varying the pH from 1 to 12, (**b**) Evolution of GQDs PL intensity when varying the pH from 1 to 12, (**c**) Photostability of GQDs (Hg/Xe lamp, intensity of 100 mW/cm^2^), and (**d**) PL intensity of GQDs in NaCl solutions with concentrations varying from 0.2 to 1 M.

The pH of the aqueous solution containing the dots was varied from 1 to 12 to further explore the optical properties of GQDs. As can be seen in Figure 6a,b, the PL intensity is the highest at pH values ranging from 4 to 8. The PL intensity remains high in acidic medium up to pH 1, but it markedly decreases at pH values that are higher than 8. This strong decrease of the PL intensity likely originates from the neutralization of ammonium into amine groups, which induces changes in defect-related electronic transitions (disruption or even prohibition). The high PL QY of GQDs at acidic pH might not only be of interest for bioimaging, but also for the development of pH-responsive drug delivery systems due to the acidic environment of inflammatory and tumor tissues.

GQDs exhibit a high photostability under the continuous irradiation of a Hg-Xe lamp (intensity of 100 mW/cm^2^). The PL intensity and the PL QY only weakly decreased remained during the irradiation (Figure 6c and Figure S4). Finally, the colloidal stability of GQDs was investigated in NaCl solutions, with concentrations varying from 0.2 to 1 M (Figure 6d). No decrease of the PL intensity was detected, which indicated that GQDs do not aggregate in a medium of high ionic strength.

### 3.3. Biocompatibility

**Figure 7:**
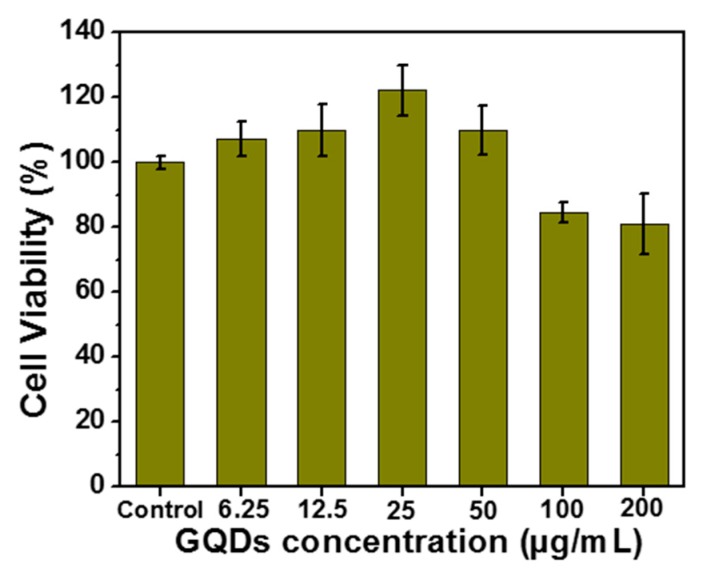
WST-1 assay on THP-1 cell line when varying the concentration of GQDs from 6.25 to 200 µg/mL.Values represent means ± S.E (*n* = 3).

The biocompatibility of GQDs was also evaluated to demonstrate that these nanoparticles can be used for biological applications without altering the cell viability. The THP-1 cell line was chosen, because circulating monocytes are considered as the first barrier of the organism against nanoparticles [69,70]. Moreover, the THP-1 cells are considered as an immune in vitro cell model and they are validated for nanotoxicological studies [71]. The WST-1 assay, which is linked to the metabolic activity of cells (mitochondrial succinate deshydrogenase function), demonstrates that the viability of the THP-1 cells only declined by less than 20% upon incubation with GQDs up to 200 µg/mL (Figure 7). The viability values above 100% that were observed for concentrations of GQDs lower than 50 µg/mL originate from the hormesis phenomenon, which is a dose response phenomenon characterized by a low dose stimulation, high dose inhibition, resulting in either a J-shaped or an inverted U-shaped dose response.

### 3.4. Antioxidant Activity

#### 3.4.1. Fe(II) and Cu(II) Chelating Properties

**Figure 8:**
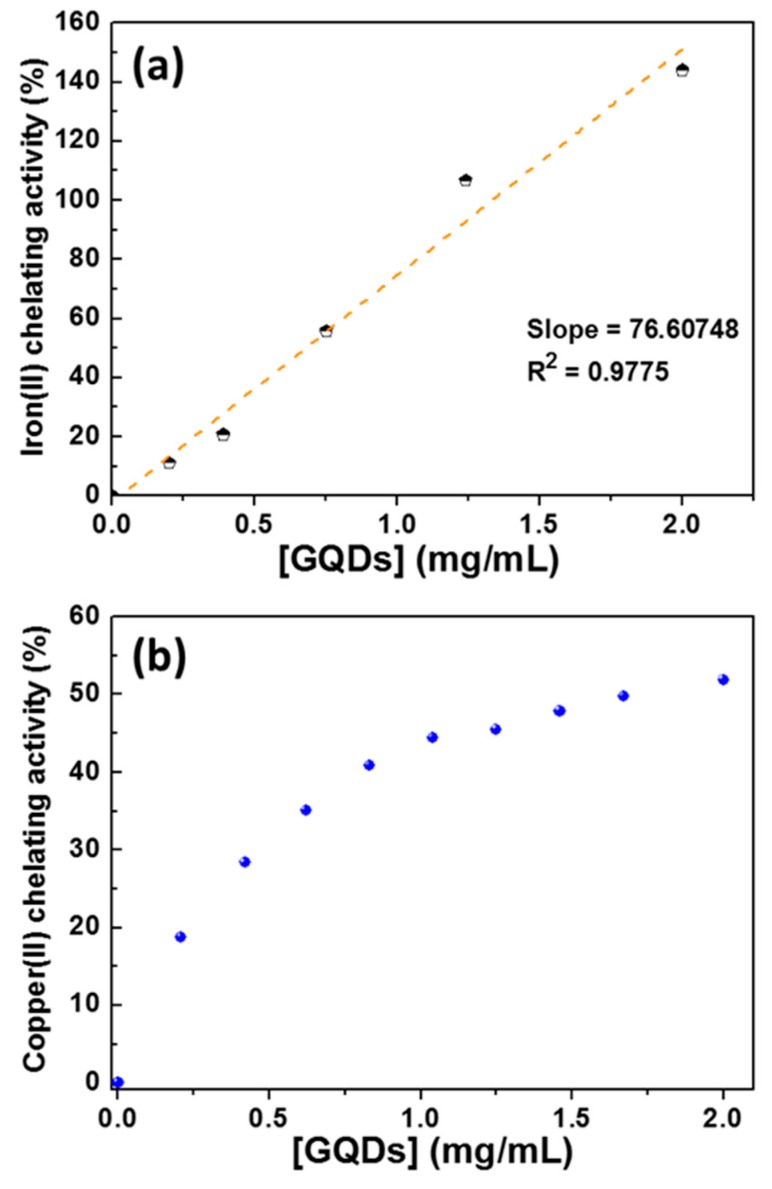
(**a**) Fe(II) and (**b**) Cu(II)-chelating abilities of GQDs.

In a first set of experiments, we evaluated the Fe(II) and the Cu(II) chelating ability of GQDs and compared the results with those that were obtained while using EDTA and carnosine, compounds that are well-known to exhibit metal chelating properties [53,54,55,56,57]. GQDs, used at various concentrations, were added to a 2 mM solution and FeCl_2_ and the mixture was incubated for 3 min. at room temperature. The amount of non-chelated Fe(II) was determined while using ferrozine which forms a complex with an UV-visible absorption maximum located at 562 nm upon complexation with Fe(II). As can be seen from Figure 8a, Fe(II) complexation linearly increases with GQDs concentration (R^2^ = 0.9775), which indicates that amine and carboxylate functions that are present at the surface of GQDs are involved in the chelation of Fe(II). When comparing these results with those that were obtained using carnosine and EDTA (Figure S5) and using Equation (1), a solution of GQDs at 0.665 mg/mL exhibits the same Fe(II) chelating activity than a carnosine solution at 471 µM or than an EDTA solution at 44.8 µM.

Similar experiments were conducted while using the Cu(II)-murexide complex to evaluate the capability of GQDs to complex Cu(II). The obtained results were found to be more complex than those that were obtained with Fe(II), with a linear increase of the Cu(II) chelating ability with the increase of GQDs concentration until ca. 1 mg/mL, followed by a levelling-off from 1 to 2 mg/mL (Figure 8b and Figure S6). For example, a solution of GQDs at 0.402 mg/mL has the same Cu(II) chelation activity than a carnosine solution at 5.69 mM or than an EDTA solution at 1.82 mM.

Fe(II) and Cu(II) are well known to promote the generation of reactive oxygen species (ROS) via the Fenton or the Haber Weiss reactions [72]. These ROS react with DNA, proteins, and membrane lipids, and they cause an oxidative stress responsible for various and pathological disorders. Thus, GQDs might be of interest to avoid the oxidative damages induced by free Fe(II) and Cu(II) metal cations due to their chelating properties.

#### 3.4.2. Radical Scavenging Capacity

**Figure 9:**
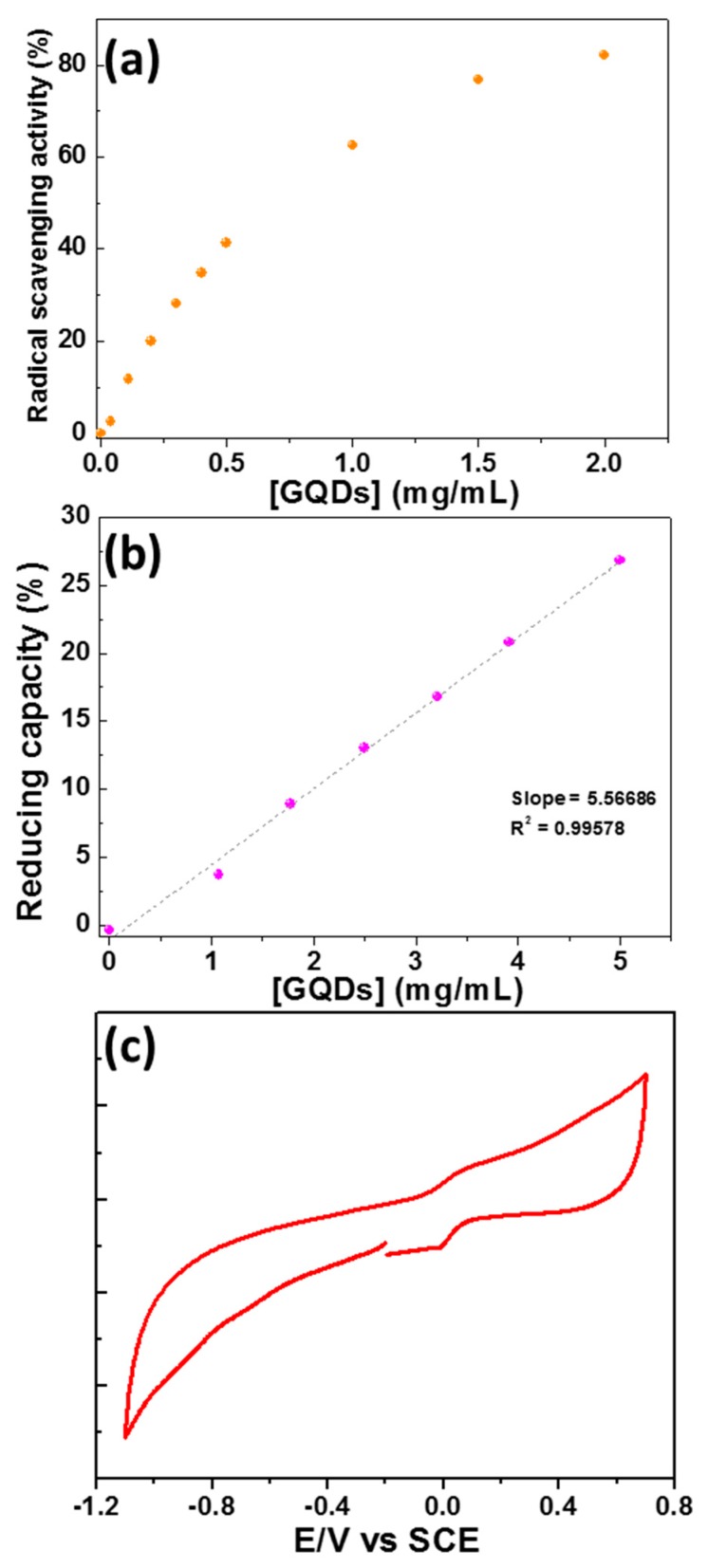
(**a**) Radical scavenging activity of GQDs, (**b**) reducing capacity of GQDs, and (**c**) cyclic voltammogram of GQDs in DMF containing 0.1 M tetrabutylammonium hexafluorophosphate (TBAPF_6_).

Next, the radical scavenging ability of GQDs was compared to Trolox (6-hydroxy-2,5,7,8-tetramethylchroman-2-carboxylic acid), a soluble analogue of vitamin E commonly used as radical scavenging reference, towards the 2,2’-azino-bis(3-ethylbenzothiazoline-6-sulfonic acid) radical (ABTS^•+^) [53,58,59]. For that purpose, the decrease of the ABTS^•+^ UV-visible absorption at 734 nm was monitored upon the addition of GQDs and the radical scavenging activity determined while using Equation (3) (Figure 9a and Figure S7). As can be seen, the radical scavenging activity linearly increases with the concentration of GQDs until 0.5 mg/mL, while the increase in activity is lower above this value. For example, while considering a 30% radical scavenging activity, a solution of GQDs at 0.342 mg/mL exhibits the same radical scavenging activity than a Trolox solution at 21.74 µM.

#### 3.4.3. Reducing Capacity

Finally, the reducing capacity of GQDs was compared to sodium ascorbate for the reduction of K_3_Fe(CN)_6_. The UV-visible absorption at 700 nm of the Prussian dye formed was monitored and the reducing capacity determined while using Equation (4) (Figure 9b and Figure S8). A linear increase with GQDs concentration is observed, which indicated that electrons could be easily transferred from GQDs to Fe(III). A reducing activity of 10% corresponds either to a 30.6 µM sodium ascorbate solution or to a 1.9 mg/mL of GQDs solution.

The redox properties of GQDs were investigated by cyclic voltammetry in deoxygenated dimethylformamide with tetrabutylammonium hexafluorophosphate (TBAPF_6_) as the supporting electrolyte in a standard three-electrode cell, being composed of a glassy carbon electrode, a platinum counter electrode, and a saturated calomel reference electrode (SCE) to ascertain their capacity to reduce [Fe(CN)_6_]^3−^. In the oxidation part, the cyclic voltammogram reveals a broad redox process, which displays some reversibility (Figure 9c). Indeed, the differential pulse voltammetry experiment (DPV) exhibits a signal at 0.02 V vs. SCE attributed to their first oxidation process (Figure S9). This value is consistent with the reductant properties that were observed for GQDs towards Fe(III).

## 4. Conclusions

A simple and efficient method for preparing 2,2’-(ethylenedioxy)bis(ethylamine)-functionalized GQDs via the oxidative fragmentation of GO is described. GQDs have an average diameter of ca. 4 nm and they emit strong green-yellow fluorescence with a PL QY of 22% at the excitation wavelength of 375 nm. Meanwhile, GQDs exhibit high colloidal and photostabilities, along with a good biocompatibility with THP-1 cells. The dots can be used to trap Fe(II) and Cu(II) cations and scavenge radicals, like ABTS^•+^, due to the radical scavenging properties of sp^2^-carbon domains and to the chelating properties of oxygen-containing functions and of 2,2’-(ethylenedioxy)bis(ethylamine) moieties present at the surface of GQDs. These results demonstrate that GQDs have high potential for bioimaging and in health applications.

## Supplementary Materials

The following are available online at https://www.mdpi.com/2079-4991/10/1/104/s1, Figure S1: ^1^H NMR spectrum of GQDs dispersed in D_2_O; Figure S2: (a) ^13^C NMR spectrum of GQDs dispersed in D_2_O and (b) magnification of the 190-80 ppm region; Figure S3: Normalized PL emission spectra of GQDs dispersed in water when varying the excitation wavelength from 300 to 500 nm with an increment of 25 nm; Figure S4: Time course evolution of the PL emission spectra of GQDs (λ_ex_ = 375 nm) during the continuous irradiation of a Hg-Xe lamp (intensity of 100 mW/cm^2^); Figure S5: Iron(II) chelating activities of (a) EDTA and (b) carnosine; Figure S6: Copper(II) chelating activities of (a) EDTA and (b) carnosine; Figure S7: Radical scavenging activity of Trolox; Figure S8: Reducing capacity of ascorbic acid, Figure S9: Differential pulse voltammogram of GQDs in DMF containing 0.1 M TBAPF_6_.
