# Supplementary material for: One-Step Synthesis of Diamine-Functionalized Graphene Quantum Dots from Graphene Oxide and Their Chelating and Antioxidant Activities
Source: Nanomaterials (Basel). 2020 Jan 4;10(1):104. doi: 10.3390/nano10010104 (PMC7023243; doi:10.3390/nano10010104)
Supplement: Supplementary file 1 [file nanomaterials-10-00104-s001.pdf]

SUPPORTING INFORMATION

Article

One-Step Synthesis of Diamine-Functionalized Graphene Quantum Dots from Graphene Oxide and Their Chelating and Antioxidant Activities

Rabeb El-Hnayn [1], Laetitia Canabady-Rochelle [2], Christophe Desmarets [3], Lavinia Balan [4,5], Hervé Rinnert [6], Olivier Joubert [6], Ghouti Medjahdi [6], Hafedh Ben Ouada [1], and Raphaël Schneider [2,*]

1 Laboratoire des Interfaces et des Matériaux Avancés, Faculté des Sciences de Monastir, Avenue de l'Environnement, 5019 Monastir, Tunisia; lahnayen1986@gmail.com (R.E.-H.); hafedhbenouada@gmail.com (H.B.O.)

2 Laboratoire Réactions et Génie des Procédés, LRGP, Université de Lorraine, CNRS, F-54000 Nancy, France; Laetitia.canabady-rochelle@univ-lorraine.fr

3 Institut Parisien de Chimie Moléculaire UMR-CNRS 8232, Sorbonne Université, 4 Place Jussieu, 75252 Paris CEDEX 5, France; christophe.desmarets@sorbonne-universite.fr

4 Institut de Science des Matériaux de Mulhouse (IS2M), CNRS, UMR 7361, 15 rue Jean Starcky, 68093 Mulhouse, France; lavinia.balan@cnrs-orleans.fr

5 CEMHTI-UPR3079 CNRS, Site Haute Température, 1D avenue de la Recherche Scientifique, 45071 Orléans, France

6 Institut Jean Lamour, Université de Lorraine, CNRS, IJL, 54506 Vandoeuvre-lès-Nancy CEDEX, France; herve.rinnert@univ-lorraine.fr (H.R.); olivier.joubert@univ-lorraine.fr (O.J.); ghouti.medjahdi@univ-lorraine.fr (G.M.)

* Correspondence: raphael.schneider@univ-lorraine.fr; Tel.: +33-3-72-74-37-90

Received: 2 December 2019; Accepted: 27 December 2019; Published: date

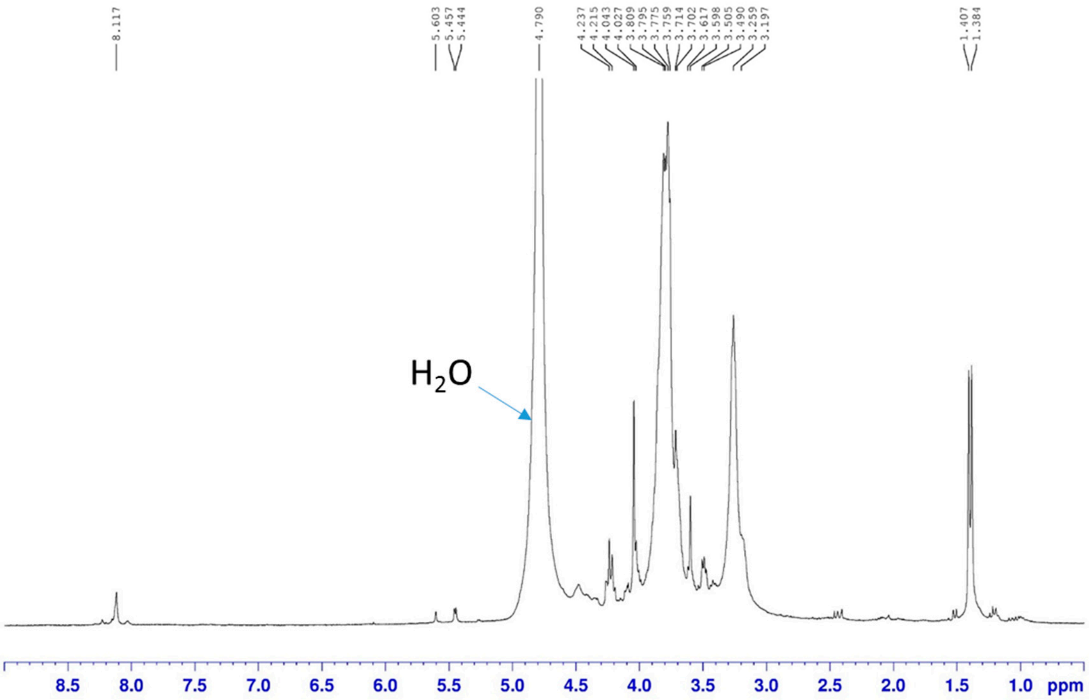

Figure S1. ^{1}H NMR spectrum of GQDs dispersed in D_2O.

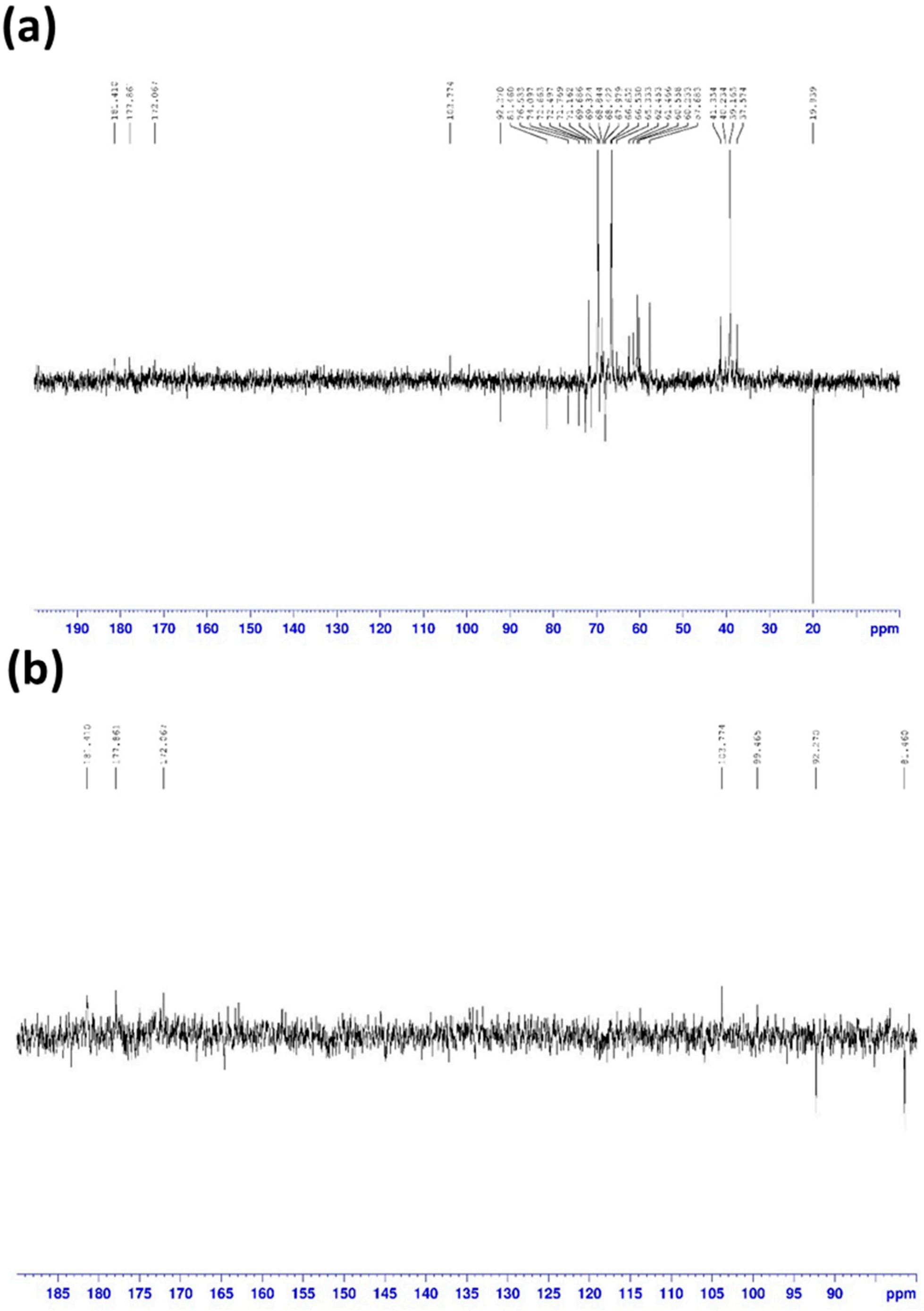

Figure S2. (a) ^{13}C NMR spectrum of GQDs dispersed in D_2O and (b) magnification of the 190-80 ppm region.

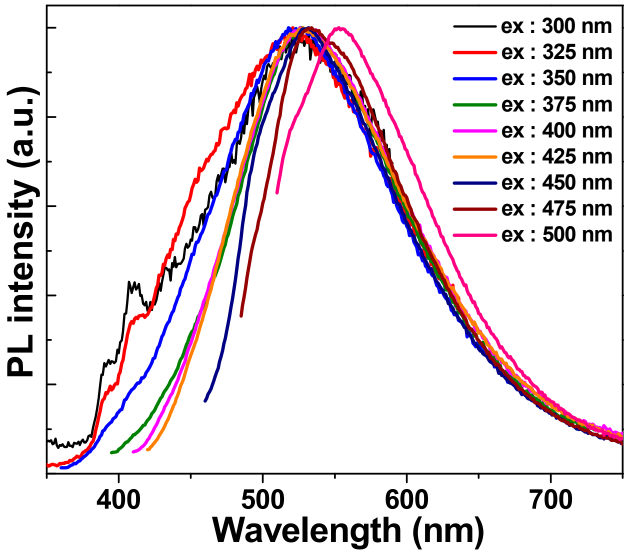

Figure S3. Normalized PL emission spectra of GQDs dispersed in water when varying the excitation wavelength from 300 to 500 nm with an increment of 25 nm.

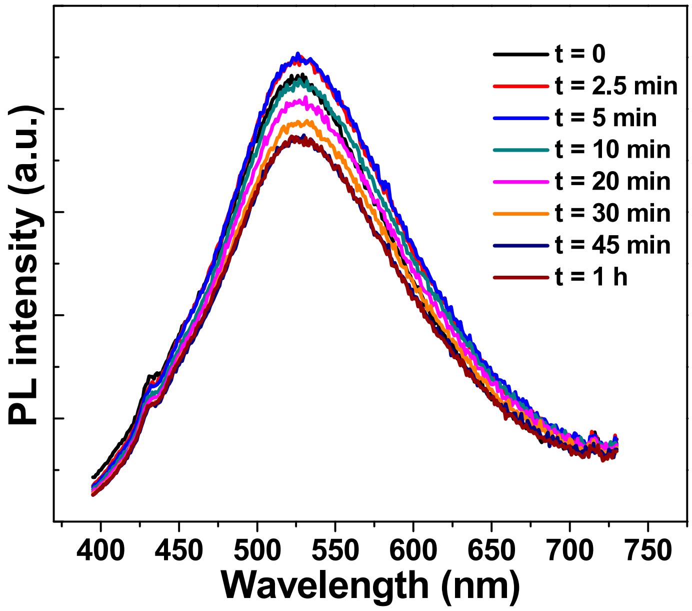

Figure S4. Temporal evolution of QDs PL emission spectra (λ_{ex} = 375 nm) during the continuous irradiation of a Hg-Xe lamp (intensity of 100 mW/cm^2).

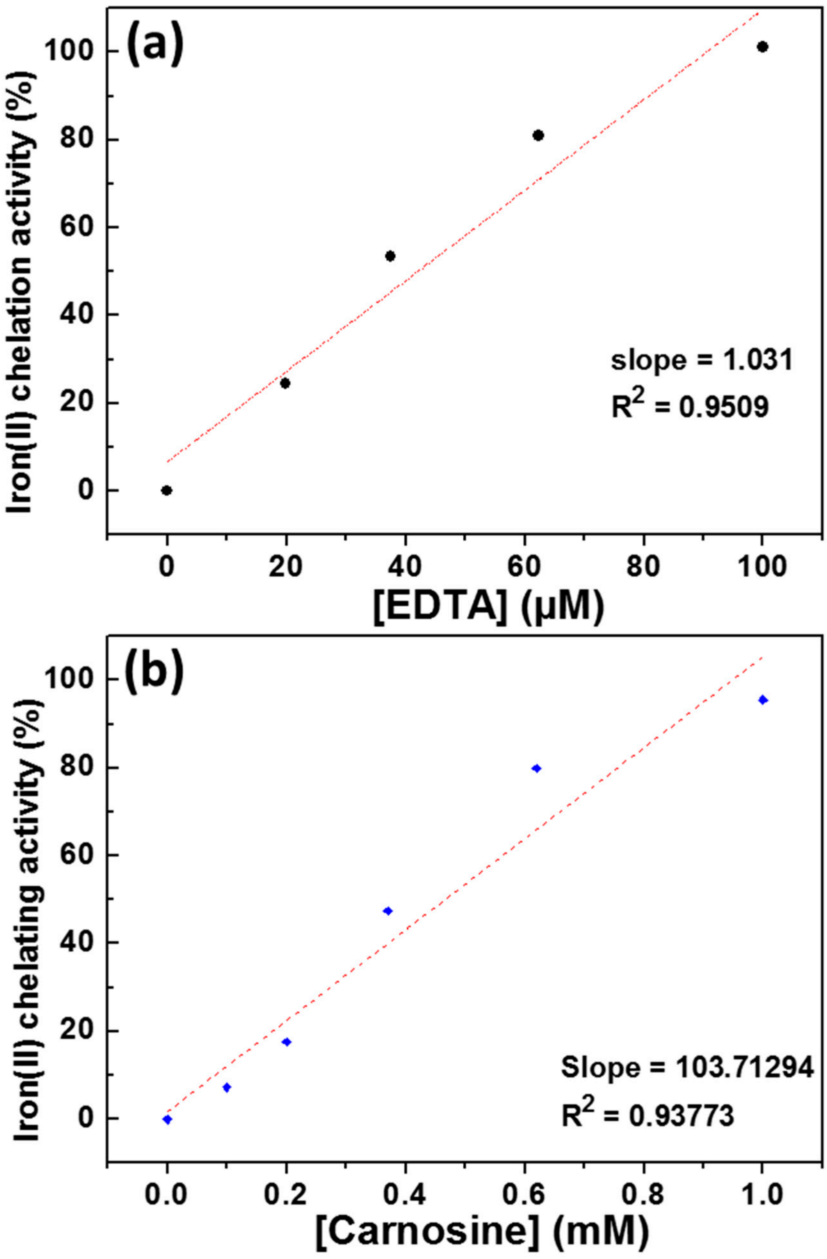

Figure S5. Iron(II) chelating activities of (a) EDTA and (b) carnosine.

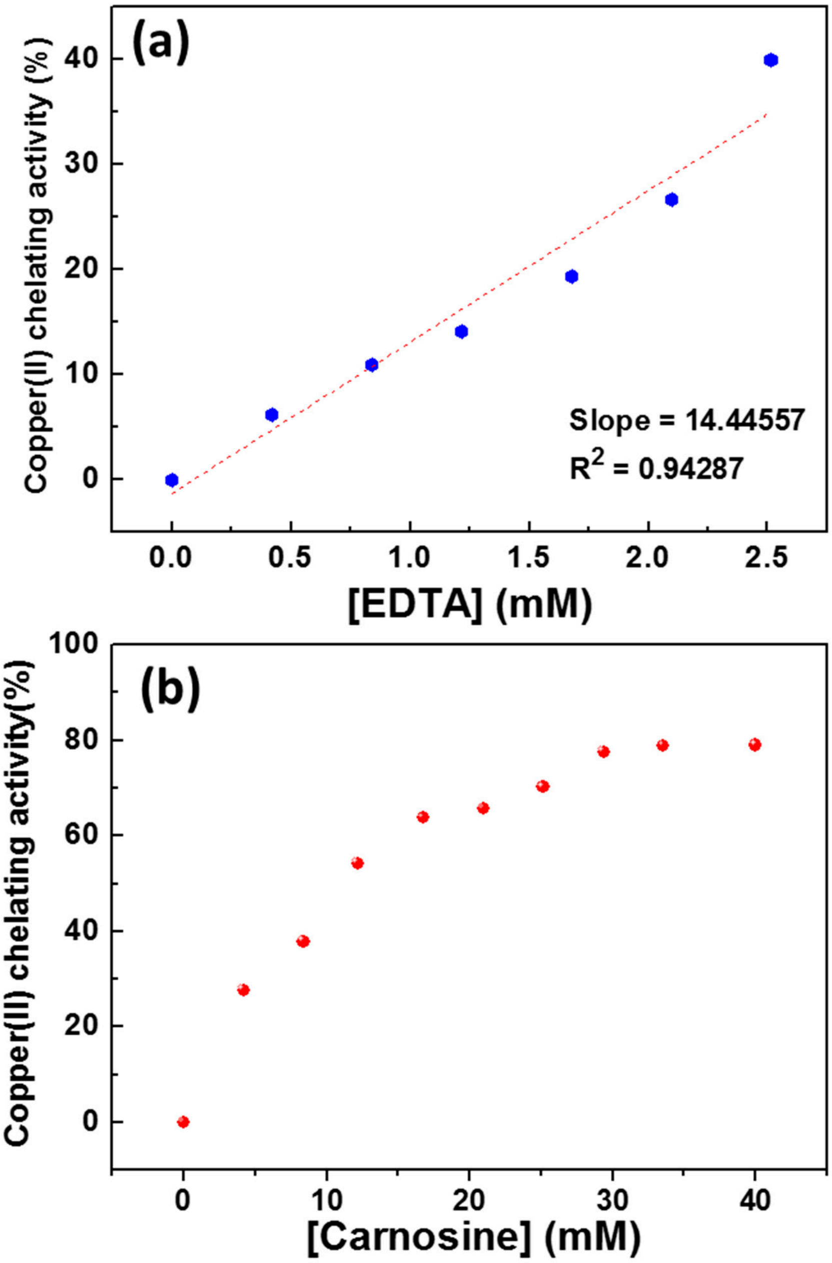

Figure S6. Copper(II) chelating activities of (a) EDTA and (b) carnosine.

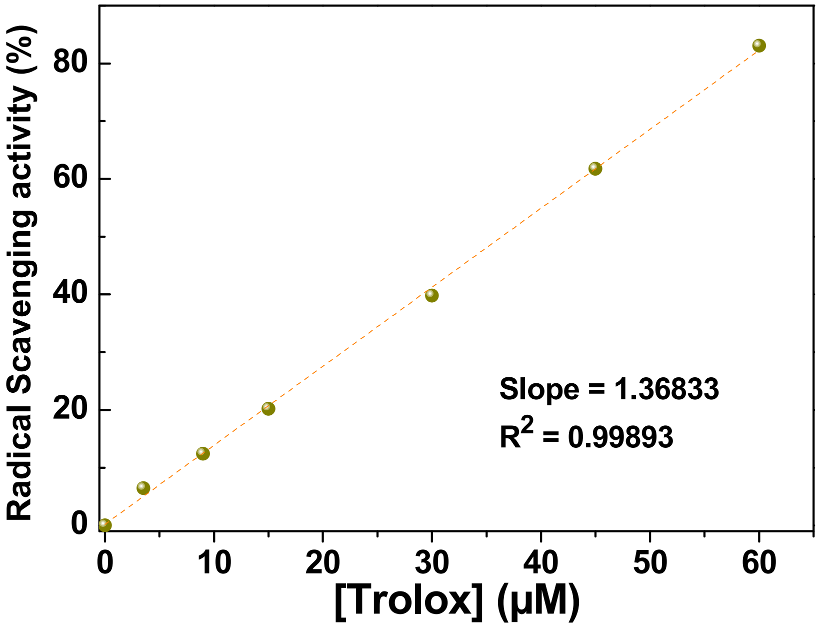

Figure S7. Radical scavenging activity of Trolox.

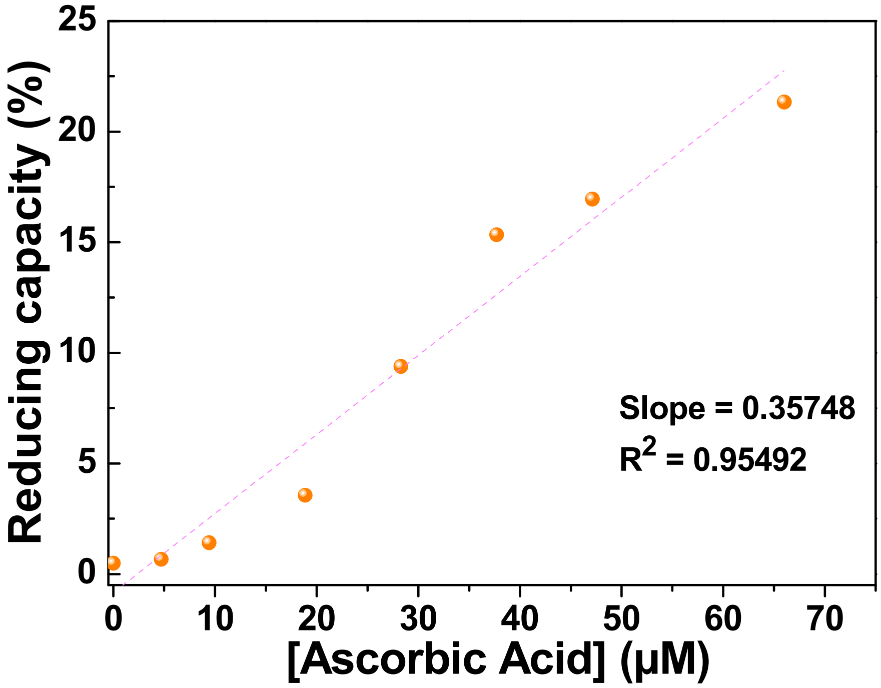

Figure S8. Reducing capacity of ascorbic acid.

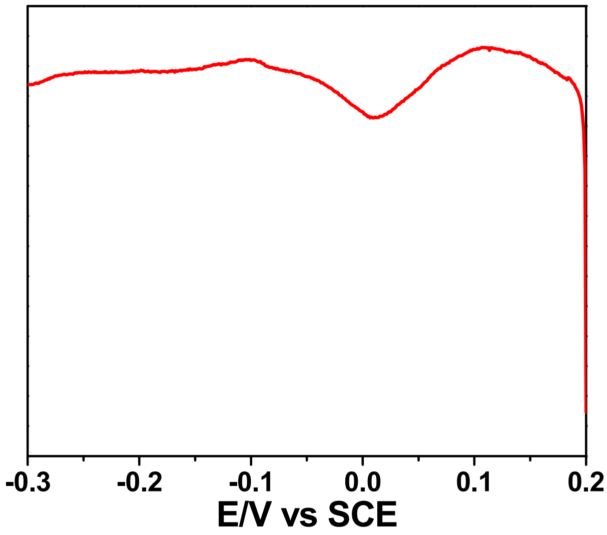

Figure S9. Differential pulse voltammogram of GQDs in DMF containing 0.1 M $TBAPF_6$.
